# Saberes médicos e práticas de cura tradicionais dos bororos: relatos de uma expedição ao sertão de Mato Grosso, 1911

**DOI:** 10.1590/S0104-59702024000100073

**Published:** 2025-01-10

**Authors:** Robson Mendonça Pereira

**Affiliations:** i Docente, Universidade Estadual de Goiás. Anápolis – GO – Brasil. robsonmenper@hotmail.com

**Keywords:** Plantas medicinais, Comissão Rondon, Intermediário, Murillo de Campos (1887-1968)

## Abstract

Este texto analisa os relatos médicos do doutor Murillo de Campos sobre uma expedição da Comissão Rondon ao Mato Grosso, em 1911. Publicado originalmente em forma de artigo científico, começa detalhando o quadro nosológico e os costumes dos habitantes das localidades visitadas. Em seguida, apresenta um denso relato de uma pesquisa de campo sobre as práticas de cura de indígenas bororos do leste de Mato Grosso. Adota uma abordagem investigativa, ao descrever meios terapêuticos utilizados pelos curandeiros e destaca especialmente o amplo emprego de plantas medicinais. O objetivo é demonstrar que Murillo de Campos agiu como intermediário entre os saberes médicos e as práticas de cura bororo.

## Percurso de um médico militar na Comissão Rondon

Em sua edição de 22 de março de 1913, a revista semanal *Brasil-Médico*, importante periódico na área de higiene pública e medicina tropical,^1^ publicou na seção “Trabalhos originais” o artigo “Notas do interior do Brasil: do Rio de Janeiro a Cuiabá (via Goiás)”, de autoria de Murillo de Souza Campos (1913b, p.111-116), médico que vinha atuando no Serviço Sanitário da Comissão Rondon, como ficou conhecida a Comissão de Linhas Telegráficas Estratégicas do Mato Grosso ao Amazonas (CLTEMTA). O autor apresenta, nesse texto, um minucioso relatório médico-científico sobre uma longa viagem, na qual acompanhava uma turma de inspeção do recém-criado Serviço de Proteção aos Índios (SPI),^2^ que teria como destino final Cuiabá, no Mato Grosso, sede principal das operações da Comissão.

Apesar de sua curta passagem pela Comissão, o resultado de sua experiência de imersão na comissão do telégrafo pode ser notado no rico manancial desse e de outros textos de sua autoria, reunidos mais tarde na obra *Interior do Brasil: notas médicas e etnográficas* (Campos, 1936).

Sobre a trajetória do médico Murillo de Campos, pode-se dizer que foi semelhante à de seus pares que passaram pela CLTEMTA. De origem paulista, nasceu em 1887, na cidade de Amparo. Estudou na Faculdade de Medicina do Rio de Janeiro, formando-se em 1908. No ano seguinte, passou a integrar o Serviço de Saúde do Exército. Em maio de 1910, o primeiro-tenente embarcou para Cuiabá e, em seguida, para o acampamento avançado da CLTEMTA, situado às margens do rio Juruena, para cumprir sua primeira missão.

Quando Campos assumiu seu posto no Serviço Sanitário, a comissão contava com três clínicos, Joaquim Augusto Tanajura, João Florentino Meira de Faria e Paulo Fernandes dos Santos. Os três desempenhavam suas atividades nos diversos acampamentos de trabalhadores espalhados ao longo da linha telegráfica em construção, de modo que percorriam enormes distâncias para atender os muitos acidentados e doentes, geralmente de malária ou de alguma enfermidade endêmica tropical. Contudo, é importante destacar que os médicos militares eram também designados para inúmeras expedições de reconhecimento.

Naquele momento, o Serviço Sanitário passou por uma reorganização interna que ampliou o campo de ação e a autonomia dos médicos da CLTEMTA, em consonância com as mudanças que vinham sendo realizadas no Serviço de Saúde do Exército (Vital, 2011, p.38-40). Um ponto fundamental nesse processo foi o de garantir que os médicos dispusessem de autoridade e de autonomia.

O chefe da Comissão Cândido Rondon incumbiu o médico Joaquim Tanajura da feitura das instruções para o novo Serviço Sanitário, que foi aprovado em 22 de maio de 1910 (CLTEMTA, [1911], p.109-113). De imediato, foram determinadas duas mudanças fundamentais: a instalação de duas enfermarias, uma em Santo Antônio do Madeira e outra na Serra do Norte; e a criação de um Serviço de Profilaxia contra o Paludismo, medida urgente e necessária para evitar surtos epidêmicos de malária, como o que chegou a paralisar os serviços de construção entre julho de 1910 e julho de 1911 (Diacon, 2006, p.81; Vital, 2011, p.69-71).

Os médicos designados para as enfermarias poderiam tomar decisões independentes do comando, como determinar o isolamento de doentes e a prescrição de doses obrigatórias diárias de quinina aos praças e civis em serviço. Além disso, o escopo da ação do serviço sanitário foi ampliado, ao fazer com que os médicos acumulassem as “funções de investigadores e analistas das doenças da região, conselheiros dos chefes das expedições, encarregados da profilaxia da malária e da cura de doentes e feridos” (Caser, Sá, 2010, p.372-373).

Nesse sentido, o médico era estimulado a agir como um cientista, podia, “a título de experiência, ensaiar o cultivo de plantas recomendadas nas zonas paludosas e de legumes para o uso do pessoal toda vez que isso for possível, ainda mesmo não podendo colher seus resultados imediatos”. Deveria também produzir observações minuciosas sobre casos de úlcera, berne, beribéri e sobre “qualquer moléstia que julgar digna de análise, estudando particularmente as suas causas e os insetos vetores porventura responsáveis pela sua transmissão” (CLTEMTA, [1911], p.111).

Um indicativo de que os médicos da CLTEMTA se preparavam para atuar como cientistas foi o de que muitos passavam por algum tipo de treinamento específico para investigarem as principais doenças tropicais da região noroeste.

O capitão-médico José Antônio Cajazeira, que foi membro da Expedição Científica Roosevelt-Rondon (1913-1914), realizou um curso completo de seis meses no Instituto Oswaldo Cruz (IOC). Foi-lhe permitido, ainda, permanecer mais cinco meses em Manguinhos para se aperfeiçoar em outros assuntos (CLTEMTA, 1916, p.4). Em seu relatório, defendia que os médicos deveriam produzir um conhecimento próprio das patologias tropicais. Aconselhava como “indispensável um microscópio pequeno, portátil, com sua caixa de reativos imprescindíveis, escolhidos e preparados pelo médico da expedição, o qual, além de estar habituado à convivência de semelhante aparelho, deve conhecer mais ou menos as moléstias dominantes na zona a explorar, a fim de poder organizar melhor sua ambulância” (p.14).

O conhecimento popular obtido no contato com os locais foi essencial para que esses médicos pudessem “realizar estudos sobre as doenças e seus ciclos no noroeste do país” (Vital, Hochman, 2013, p.78). Além disso, contribuiu para que pudessem investigar as particularidades locais de moléstias que assolavam os trabalhadores, como a malária, a ancilostomíase e o beribéri. Na maioria das vezes, só podiam contar com os saberes dos sertanejos e dos indígenas, que eram frequentemente consultados. Juntam-se a isso as observações acerca dos “ciclos de cheias dos rios e aos fluxos migratórios” (p.78) para composição de um quadro epidemiológico com o qual se pudessem “definir estratégias, baseadas na medicina tropical, a fim de evitar graves epidemias entre os trabalhadores da Comissão” (p.78-79).

Para uma melhor compreensão sobre a maneira pela qual esses médicos adquiriam conhecimento a respeito de “eventos, lugares e pessoas poucos conhecidos” (Latour, 2000, p.362), recorro à definição de Bruno Latour de que esse processo cognitivo somente seria possível “por meio do exame de todo um ciclo de acumulação” (p.357) de conhecimento. Os médicos da Comissão, como observadores atuando a distância, recorriam aos mesmos mecanismos desenvolvidos pelos sanitaristas e epidemiologistas para efetuar suas pesquisas de campo em locais remotos do país.

Em suas próprias expedições de reconhecimento, Rondon também se fez acompanhar por cientistas. O médico designado para cuidar do serviço sanitário, assim como alguns oficiais, frequentemente se envolvia em atividades de coleta de plantas, insetos e animais, adquirindo um aprendizado na manipulação de espécimes.

Em julho de 1910, Murillo de Campos embarcou em sua primeira missão como membro de uma expedição exploradora de estudo da flora do rio Juruena. Organizada por Rondon, a pedido do botânico Frederico Carlos Hoehne, a expedição foi chefiada pelo capitão Manuel Teóphilo da Costa Pinheiro. Na ocasião, Campos colaborou no trabalho de coleta de material zoológico, etnográfico e da flora, empreendido por Hoehne, para o Museu Nacional do Rio de Janeiro (Sá, Sá, Lima, 2008, p.794).

Campos (1913a) publicou o resultado desse seu trabalho investigativo em outro artigo, intitulado “Notas do interior do Brasil: noroeste de Mato Grosso”, na revista *Arquivos Brasileiros de Medicina*. No texto, apresenta um quadro da flora medicinal sertaneja e indígena da região e um levantamento das enfermidades e práticas de cura entre os povos pareci e nambiquara. Percebe-se, assim, um diálogo entre esse artigo e o seu homônimo na *Brasil-Médico*. Em ambos os textos, nota-se que o autor dependeu de diversos colaboradores, entre os habitantes locais (seringueiros, indígenas etc.), os quais lhe forneceram informações importantes acerca das moléstias reinantes nas paragens percorridas, assim como sobre os saberes e as práticas de cura tradicionais.

Nesse sentido, Campos, assim como outros médicos da comissão, era capaz de atuar como uma espécie de intermediário na acepção conferida por Kapil Raj (2007). Isto é, como indivíduo com capacidade para efetuar algum tipo de mediação, seja como intérprete ou tradutor, no ponto de encontro entre dois mundos; neste caso, o da medicina científica e o do saber popular tradicional indígena (Antunes, 2019, p.43).

Os médicos da comissão constituíam, assim, um grupo intermediário especializado, capaz de avaliar os conhecimentos de práticas. Esse conhecimento de práticas dependia necessariamente de uma negociação entre especialistas e colaboradores locais para sua elaboração e certificação (Raj, 2007, p.14). Trata-se de conhecimento construído, segundo Callon, Lascoumes e Barthe (2009), num espaço “ao ar livre” (*recherche de plein air*), o qual possui determinadas especificidades em relação àqueles produzidos pela ciência de campo ou nos laboratórios.

### Relatos de uma viagem de inspeção do Serviço de Proteção aos Índios

A jornada do médico Murillo de Campos rumo ao acampamento da CLTEMTA teve início no Rio de Janeiro, quando embarcou em trem da Estrada de Ferro Central do Brasil para São Paulo, em maio de 1911. Da capital, seguiu viagem pelo interior até a vila de Bauru. Dali partiam os trilhos da Noroeste do Brasil, ferrovia que permitiu o acesso a uma vasta porção do território paulista que se abriu à ocupação fundiária predatória. A região noroeste foi cartografada, nessa mesma época, pela Comissão Geográfica e Geológica de São Paulo, e representada nos mapas como se fosse um espaço vazio e desconhecido. Essa estratégia permitiu a legitimação do apossamento das terras públicas por grileiros, que depois as negociaram com os cafeicultores (Arruda, 2000, p.123-124; Dean, 1996, p.229-230). Esse processo acirrou os conflitos entre colonos brancos e os indígenas, que foram massacrados ou deslocados para aldeamentos oficiais (Mota, Schraiber, 2021, p.440-445).

Campos (1913b, p.111) descreve a paisagem e as condições ambientais que favoreciam a reprodução do vetor da malária. O mosquito *Anopheles*, justamente no trecho mais extenso da ferrovia, expunha os trabalhadores envolvidos nas obras de construção à doença e à morte.

A percepção de que as endemias que reinavam no noroeste paulista colaboravam para deteriorar a vida operária na região fora notada por Arthur Neiva, pesquisador do IOC contratado pela ferrovia para “combater a malária nos canteiros de obras” (Benchimol, Jogas Junior, 2020, p.45). Neiva considerava que a exploração dos trabalhadores, a invasão de terras indígenas e o avanço sobre as florestas havia colaborado especialmente na “propagação de forma rápida e profunda” da leishmaniose. Conhecida na região como “úlcera de Bauru”, seu primeiro diagnóstico na América Latina foi realizado em 1909, por pesquisadores do Instituto Pasteur e do Instituto Bacteriológico paulista, no momento em que havia um intenso debate internacional sobre o tema (Mota, Schraiber, 2021, p.445-446; Benchimol, Jogas Junior, 2020, p.13).

Ao descer na estação de Itapura, ponto final da ferrovia situada próxima à foz do rio Tietê, Campos realizou exames em soldados de um contingente do Batalhão de Caçadores e nos índios Kaingang que viviam próximos a um posto do SPI. Além da prevalência dos casos de malária, registrou também outros de natureza endêmica, como a ancilostomíase, o bócio, a sífilis e o alcoolismo. Em relação aos remanescentes dos Kaingang, menciona, mesmo que de forma ligeira, a condição miserável em que se encontravam. Resistiram ao avanço das obras de construção da ferrovia que usurparam seu território, mas foram dizimados e tiveram suas aldeias destruídas por bugreiros contratados pela empresa e por posseiros (Ghirardello, 2002, p.42-43).

Campos comenta ainda sobre a ausência de incidência do beribéri, reportada por médicos que atuaram durante a construção do trecho paulista da ferrovia (1905-1910), em contraste com a região noroeste de Mato Grosso, onde a moléstia era observada constantemente, fato que atribuiu a diferenças na qualidade da alimentação dos operários (Ghirardello, 2002, p.40, 44). É notável também a ausência de qualquer registro de caso de febre amarela e de leishmaniose, principalmente desta segunda, cuja incidência se manteve alta na zona noroeste ao longo da década de 1910. Essas moléstias eram consideradas importantes nos estudos do quadro epidemiológico das regiões Centro-Oeste e Amazônica desenvolvidos pelo IOC (Ghirardello, 2002, p.41; Lima, 2013, p.137-138; Benchimol, Jogas Junior, 2020, p.44-45).

Na travessia do Triângulo Mineiro, ao passar pela localidade de Uberaba, Campos percebe que seus habitantes apresentavam moléstias similares às encontradas no noroeste paulista. Além dos casos de lepra, alude brevemente a duas enfermidades cutâneas com base em informações dos locais: o “fogo selvagem” (pênfigo foliáceo), de causa desconhecida, e a *caphobira* (cafubira), “que parece estar ligada à sífilis” (Campos, 1913b, p.111). Comenta que havia encontrado habitações infestadas de barbeiros em fazenda próxima à vila de Araguari. Essa menção é significativa, pois, na época, se iniciava uma extensa busca pelo interior do país por triatomíneos. Estes eram enviados para os laboratórios de institutos como Manguinhos, para ser examinados. Verificava-se, assim, se o *Trypanossoma cruzi*, causador da moléstia de Chagas (tripanossomíase brasileira), era encontrado (Silva, 2021, p.124-125). Vital e Hochman (2013, p.89) assinalam que talvez Murillo de Campos compartilhasse da hipótese de Carlos Chagas segundo a qual o bócio endêmico nas áreas rurais do Brasil seria uma “manifestação clínica da tripanossomíase”, diferente do bócio entre habitantes europeus.

Depois de transpor o rio Paranaíba, Campos começou a percorrer diversas localidades no interior de Goiás. Sua avaliação a respeito dos hábitos dos sertanejos tem um tom muito mais severo, ao descrever um cenário no qual o abandono da população pelo poder público favorecia um estado de permanente insalubridade:

Os goianos isolados do resto da nação, rotineiros e sem instrução, vivem disseminados ao longo das estradas de rodagem, constituindo poucas vilas. A sua habitação é, em geral, mal-acabada e anti-higiênica. Situada à beira da mata, em pequena área derribada, fica sempre próxima à água. Tem habitualmente como apêndices o paiol, o chiqueiro e o galinheiro e à roda o curral. Em consequência desse regímen anti-higiênico, ao anoitecer, voam para o interior das casas culicídios, e segundo no informaram, também os barbeiros; o bicho-do-pé e os carrapatos são encontrados comumente (Campos, 1913b, p.111).

É importante assinalar que é durante as décadas de 1910 e 1920, no âmbito da “campanha de saneamento rural” do Brasil, que começa a se fortalecer no discurso médico um consenso a respeito da necessidade de ações estatais para combater as diversas endemias. Esse movimento visava integrar e curar o sertanejo na comunidade nacional que era constituída. Foram propostos um diagnóstico das mazelas sociais e um conjunto de ações centralizadas por parte do governo federal, destinadas a intervir no espaço rural, em rejeição aos determinismos racial e climático predominantes na primeira fase das reformas sanitárias (Hochman, 1998, p.91-93).

Médicos da Comissão Rondon, como Murillo de Campos, encontravam-se, conforme Vieira (2022), em situação privilegiada para a realização de estudos clínicos das endemias rurais. Mesmo atuando em região considerada periférica, do ponto de vista da ciência nacional, o resultado de suas pesquisas abastecia com elementos originais uma “rede científica” constituída em grandes centros, como o IOC, cujos pesquisadores estudavam as mesmas doenças (Vieira, 2022, p.17-18; Schweickardt, 2009, p.26-28). Esse conhecimento produzido “em trânsito” surgia da interação entre comunidades heterogêneas de especialistas em zonas de circulação interculturais, que promoviam transformações e reconfigurações de diferentes práticas e conhecimentos (Raj, 2007; Silva, 2021; Gesteira, 2022, p.41, 55).

Alguns médicos da CLTEMTA se ocuparam da descrição pormenorizada “dos costumes, das percepções e dos saberes” comuns entre esses sertanejos (Vital, Hochman, 2013, p.86). Havia aqueles que teciam críticas sobre a maneira como “lidavam com as enfermidades” (p.86). Discorriam a respeito da inocuidade das práticas de cura das benzedeiras, raizeiros, curandeiros e práticos, enquanto outros evitavam emitir juízos, procuravam apontar para a “necessidade de atuação do Estado na educação dos habitantes” (Vital, Hochman, 2013, p.86).

Nesse aspecto, as observações e os comentários de Murillo de Campos oscilavam entre essas duas posições. No caso da primeira, um trecho de seu relatório avalia bastante depreciativamente as populações que encontrou nos estados de Goiás e Mato Grosso, entendidas, assim, como espécies de “repositórios vazios e associados à ignorância, à doença e ao arcaico” (Silva, 2021, p.197), nos quais o médico deveria intervir (Campos, 1913b, p.112).

No caso da segunda posição, demonstra interesse em entender como certos males eram compreendidos localmente. Um exemplo disso pode ser percebido quando trata da curiosa explicação corrente entre os goianos sobre a “origem hídrica” da papeira, segundo a qual certas fontes poderiam ser responsáveis por produzi-la, e outras teriam a propriedade de reduzi-la. Na época, a etiologia do bócio era desconhecida, apesar de algumas hipóteses médicas a relacionarem “à má qualidade da água, à hereditariedade e à carência de iodo no organismo humano” (Silva, 2021, p.11). Assinala que sintomas como a anemia e a febre irregular, comuns a diferentes doenças endêmicas, geravam dúvidas entre os esculápios, que acabavam recorrendo mesmo aos saberes populares, às mezinhas e garrafadas feitas com plantas medicinais do Cerrado.

Ao atravessar o rio Araguaia, a expedição seguiu rumo ao leste de Mato Grosso. A primeira impressão de Murillo de Campos a respeito daquele sertão foi a de ser menos habitado que o Sul goiano. Apresentava, porém, os mesmos “costumes e a mesma deficiência higiênica domiciliar e individual” (Campos, 1913b, p.112). Entre as inúmeras moléstias apresentadas pelos pacientes que atendeu, destaca os casos de bócio, malária, ancilostomíase, lepra, sífilis, gonorreia e beribéri.

Campos considerava o beribéri uma moléstia mais significativa que a malária, em termos de seu impacto entre os “homens por ele examinados, que incluíam índios Pareci e Nambiquara, seringueiros e trabalhadores da CLTEMTA” (Caser, Sá, 2011, p.495). Levantou a hipótese de que a incidência do beribéri na região noroeste estaria relacionada à deficiente produção agrícola, que dificultava o abastecimento de gêneros alimentares nas guarnições militares e em comissões científicas e de engenharia no sertão, que levavam à sua importação do Rio Grande do Sul ou da Argentina. Devido ao “acondicionamento e pela demora da viagem”, esses gêneros chegavam poucas vezes em “bom estado de conservação” nos acampamentos (Campos, 1936, p.73). Essa seria a causa das inúmeras manifestações da moléstia, fosse na forma endêmica, como ocorrera nas guarnições do Forte de Coimbra e de Corumbá, fosse na epidêmica, durante a construção da E.F. Noroeste do Brasil (1909) e nos trabalhos da CLTEMTA (1910) (Campos, 1913b, p.112-113).

De fato, presenciou um surto epidêmico de beribéri que atingiu quase metade do contingente de trabalhadores da Comissão, no acampamento do Juruena, entre abril e junho de 1910. Inclusive dedicou parte considerável do artigo “Noroeste de Mato Grosso” a uma minuciosa avaliação dos doentes e construiu diversas hipóteses para explicar quais fatores atuariam para tornar aquele mal tão frequente e grave (Campos, 1913b, 1936).

Na sua avaliação acerca das condições sanitárias insatisfatórias, atribui mais peso aos hábitos individuais de higiene que às condições de insalubridade do meio, defendendo a necessidade de intervenção naquela realidade social:

A impressão dominante que se tem das populações sertanejas é que seus males provêm antes da sua ignorância e negligência que da decantada insalubridade climática. A criação de um serviço de higiene rural, tendo por fim mais repisar certas verdades de higiene individual e domiciliar, mais que a modificação conveniente do meio, bela utopia em nossos tempos, seria uma medida altamente humanitária (Campos, 1913b, p.113).

Tende, assim, a aproximar-se do diagnóstico formulado por Belisário Penna e Arthur Neiva em seu relatório da expedição científica do IOC, realizada em 1912, quando percorreram diversas localidades no Nordeste e parte de Goiás (Vital, Hochman, 2013, p.89). No “retrato” que fazem do país, consideram que seria a doença, e não o clima ou a raça, o principal entrave para o progresso das regiões (Lima, 2013, p.139-140). Nesse sentido, as pesquisas em bacteriologia e sobre as patologias de moléstias tropicais necessitavam ser complementadas por projetos de saneamento que agissem no intuito de moralizar os hábitos, orientar os “costumes alimentares e higiênicos”, controlar os desvios e evitar a “degeneração”, disciplinando as práticas sexuais (Schwarcz, 1993, p.297).

### As colônias de índios bororos entre projetos ideológicos divergentes

A mudança no discurso de Campos ocorreu durante sua passagem pelas cinco colônias de índios bororos.^3^ Situadas no leste de Mato Grosso, eram constituídas de duas colônias autônomas (São Lourenço e Córrego Grande) e outras três dirigidas por missionários salesianos (Barreiros, Rio dos Tachos e Sangradouro). Os registros e impressões colhidas sobre os bororos ocupam pelo menos metade do artigo, indicando a importância conferida pelo autor a esse contato. Discorre acerca de seus costumes e hábitos alimentares, sua noção de doença, os métodos terapêuticos e recursos preventivos empregados pelos curandeiros.

O desafio de Campos parece ser o entendimento das distinções e características do quadro nosológico indígena no Mato Grosso, estudo que teve início em uma investigação anterior sobre as etnias pareci e nambiquara (Campos, 1936, p.33-58). Ao mesmo tempo, contribuiu fornecendo possibilidades para uma avaliação das prováveis propriedades medicamentosas contidas nas plantas medicinais do Cerrado (Vital, Hochman, 2013, p.93). Esses estudos desenvolvidos pelos médicos da Comissão conciliavam-se com o esforço médico-científico da nascente medicina tropical brasileira (Lima, 2013, p.131-141).

A narrativa se inicia com uma descrição dos bororos como “numerosa tribo, parte civilizada e parte ainda em estado selvagem” (Campos, 1913b, p.113). No entanto, tal distinção, além de seu viés etnocêntrico, vincula-se à perspectiva de Rondon sobre o contato com os povos indígenas, para quem haveria um “roteiro positivista do progresso da nação” (Tacca, 2001, p.26). Nesse sentido, o artigo de Campos corrobora a perspectiva oficial da Comissão de evitar mencionar a presença dos missionários salesianos que mantinham um contato intenso com os bororos desde a instalação das primeiras colônias no início do século XX (Tacca, 2001, p.26-27).

Os salesianos, que chegaram ao Brasil em 1883, decidiram abrir missões em Mato Grosso em 1894, quando organizaram uma expedição chefiada pelo padre Lassagna. O governador Manoel Murtinho entregou-lhes uma colônia de índios bororos (antiga colônia militar Teresa Cristina). Entretanto, os salesianos foram retirados da colônia sob o “argumento de que em três anos não haviam feito nada” (Novaes, 1993, p.151).

Em dezembro de 1901, 14 missionários criaram, em local chamado Barreiros, próximo ao Ribeirão dos Tachos (oitenta léguas de Cuiabá), a Colônia do Sagrado Coração de Jesus (atual Meruri). Os bororos só apareceram oito meses depois, em agosto de 1902, quando construíram uma nova aldeia, de forma que ali se instalaram 140 índios (Novaes, 1993, p.152-153). A iniciativa se ampliou em 1906, com a fundação, às margens do rio Sangradouro da colônia São José, de uma área de cerca de vinte mil hectares, com quinhentas cabeças de gado, cem muares, cinquenta ovelhas e uma casa de alvenaria. O número de índios cresceu continuamente ao longo dos anos: em 1908, havia 67 indivíduos; em 1910, trezentos, e, em 1911, 374 (p.155).

É preciso aludir ao fato de que Cândido Rondon, chefe da CLTEMTA, conhecia os bororos desde antes de sua entrada nas comissões do telégrafo. Nascido em Mimoso, no estado de Mato Grosso, em 1865, tinha, por linha materna, ascendência bororo e terena, e seu contato desde a infância com esses povos indígenas (Viveiros, 1958, p.17-18) facilitou o aprendizado da língua e de seus costumes e o influenciou em sua carreira como militar. No início dos anos 1890, passou a integrar a Comissão Construtora de Linhas Telegráficas de Cuiabá ao Araguaia, ocasião em que construíram uma estação telegráfica próxima a aldeamentos bororos do vale do rio São Lourenço.

Em 1901, quando Rondon assumiu as operações da Comissão do Telégrafo no Mato Grosso e enfrentava restrições de recursos e falta de pessoal ocasionada pela malária e por constantes deserções, convenceu o chefe bororo (*boé e-iméjera*) Oarine Ecurue e o pajé Barú, do aldeamento de São Lourenço, a ceder uma turma de 150 índios que fariam a limpeza da picada, trabalho que a remoção de troncos, deixados pela derrubada, tornava penoso. Isso ocorreria para a construção do braço da linha ligando Coxim a São Lourenço, ocasião em que os bororos ficaram submetidos à disciplina militar do acampamento (Viveiros, 1958, p.130-131; Tacca, 2001, p.24). Rondon procurou demarcar terras e intimar fazendeiros acusados de atentados contra os bororos, em orientação contrária à do governo de Mato Grosso na época, “que criara legislação estadual de colonização que deixava as terras indígenas à mercê de especuladores” (Urquiza, 2007, p.73).

Desse exemplo, podemos inferir acerca da lógica que passou a presidir as relações entre a CLTEMTA e os diversos povos indígenas ao longo da linha. Positivistas, como Teixeira Mendes e o próprio Cândido Rondon, criticavam a ação das missões religiosas, por considerarem que a conversão forçada aos valores cristãos violava as leis evolucionistas. Elaboraram um programa que ambicionava “governar as relações com os povos indígenas” (Diacon, 2006, p.122), com base na estratégia de proteção e assimilação por meio de sua pacificação.

Em junho de 1911, Rondon inspecionou as colônias indígenas a cargo dos padres salesianos no rio das Garças e no rio Barreiro (CLTEMTA, 1915, p.16). Naquela ocasião, assistiu a um ritual funerário bororo (*ítaga*) e anunciou a intenção de fundar uma povoação indígena (“Borória”) no local da velha colônia de São Lourenço, com a ideia de convertê-la em centro agrícola (Viveiros, 1958, p.342-343). Essa era parte de uma estratégia do SPI para transformar o índio em trabalhador nacional.

Os salesianos, por seu lado, vinham tentando, com pouco sucesso, integrar os bororos por meio do trabalho agrícola e afastá-los de seus antigos costumes. Lévi-Strauss (1996) comenta que os missionários, ao perceberem a importância que a disposição tradicional circular de suas aldeias tinha nos seus sistemas social e religioso, convenceram os bororos a trocar sua aldeia por outra, “onde as casas são colocadas em fileiras paralelas” (p.233). Isso alterou a estrutura arquitetônica das cabanas, suprimiu a casa dos homens (*bái mána gejéwu*), local para realização das cerimônias públicas, e colocou no seu lugar uma cruz (p.232-233).

Murillo de Campos (1913b, p.113) comenta sucintamente sobre os costumes e aspectos da cultura material bororo. Começa ao tratar de sua preferência por construírem suas aldeias próximas aos cursos d’água (ao contrário dos nambiquaras e parecis, que habitavam o planalto), de seus ranchos serem amplos, bem construídos e abrigarem de três a quatro famílias em média. Eram pouco limpos em seu interior, em contraste com o terreiro ou “bororó” (pátio da aldeia). O formato circular da aldeia bororo é “o modelo ideal para representarem a sua sociedade e seu universo” (Novaes, 1986, p.77), de maneira que uma linha diametral divide a população em dois grupos: os *eceráe*, ao norte, e os *tugarége*, ao sul. A população das aldeias é organizada em clãs: “Grupos familiares que se julgaram parentes pelas mulheres, a partir de um antepassado comum de natureza mitológica” (Ribeiro, 2006, p.29). No centro do pátio da aldeia se destaca a casa dos homens (*bái mána gejéwu*), uma cabana retangular, residência provisória de homens casados e solteiros, local reservado a cerimônias dos chefes dos clãs (Ribeiro, 2006, p.30).

Quanto aos seus hábitos alimentares, praticavam caça e pesca, além de uma pequena agricultura limitada a milho e mandioca, que era complementada por frutos do Cerrado como o palmito, o coco, os bulbos de gravatá, o mel e o suco fermentado de *acory* e do buriti.

### Práticas de cura entre os índios bororos

Ao adentrar a terapêutica bororo, Campos assinala ser sua prática um “privilégio de casta”. Existem, porém, alguns “médicos *jorubocuro* que a praticam em virtude de sua inteligência e observação” (Campos, 1913b, p.114). O *jorubocuro* ou *jorubo cure* seria um tipo de curandeiro, que é introduzido desde criança pelo seu pai no “segredo das doenças e no conhecimento dos remédios” (p.114). O *jorubocuro* emprega seus recursos visando à prevenção das moléstias (profilaxia) e ao seu tratamento, que pode ser específico ou uma panaceia. Sobre a noção de doença (*djorubo*), anota que “não passa dos seus sintomas mais aparentes: dor, febre, tosse, vômito etc.” (Campos, 1936, p.144), cuja causa é de ordem sobrenatural e influência estranha. Segundo Hartmann (1968, p.27, 30), a palavra *jorubo cure* designa remédio ou substância vegetal, decorre do aposto *erúbo* e suas variantes *orúbo* e *jorúbo*, e é frequentemente encontrado na nomenclatura botânica bororo e empregado para “aquelas plantas usadas na medicina profilática e curativa” (Hartmann, 1968, p.30).

Existem, evidentemente, algumas imprecisões na descrição feita por Campos das práticas de cura bororo, uma vez que teve de limitar o campo de sua investigação às moléstias observadas entre os pacientes indígenas que atendeu nas colônias visitadas. Ainda assim, teve tempo para colher informações acuradas sobre costumes e procedimentos por parte dos curadores dentro do limitado conhecimento etnológico da época.

Para Viertler (1987), há três tipos de curadores ou xamãs bororos responsáveis pela integridade da comunidade: o *Bari*, xamã dos espíritos; o *Aroe Etawara Are*, xamã das almas; e o *Erubudobu Epa*, senhor dos remédios. Os dois primeiros obtêm seus poderes “a partir do contato individual extraordinário com o sobrenatural” (p.207-208), e a função que lhes é atribuída se trata de “assunto de ofício, e não de clã ou nascimento” (p.207). Na cosmogonia bororo, o *Bari* e o *Aroe Etawara Are* se associam a dois tipos de forças distintas; o primeiro, aos *Bope* (“espíritos da natureza associados com determinadas plantas e animais”); e o segundo, aos *Aroe* (“espíritos aquáticos e almas de mortos”). Representam uma espécie de dualismo que remete a um passado mítico no qual teria ocorrido a fusão da antiga população de lavradores com outra mais recente de guerreiros caçadores. O *Erubudobu Epa*, por sua parte, associa-se ao *Erubo-mage*, que são “forças mágicas associadas a certos vegetais do campo e da floresta empregados como remédios ou venenos” (Viertler, 1987, p.207).

O *Bari* e o *Aroe Etawara Are* promovem a cura de doenças ao realizar a “extração, por meio de sucções, de corpos estranhos” (Albisetti, Venturelli, 1962, p.115), causadores da moléstia que se faz pela “‘ingestão de fumaça’ dos charutos sempre ofertados aos curadores associados a fortes salivações” (p.239; destaque no original). Já o curador *Erubudobu Epa* (“senhor dos remédios”) atuaria de maneira sigilosa, por meio da ingestão ou aproximação física ou simbólica do doente com alguma substância de origem vegetal, podendo também lançar feitiços contra inimigos.

É possível que esse último curandeiro ou xamã bororo seja o médico *jorubocuro* ao qual se refere Campos, por se tratar de informante privilegiado que detinha o conhecimento ancestral sobre as plantas medicinais do Cerrado e sua manipulação. O tratamento e as prescrições curativas variavam em função da opinião de cada pajé e dos supostos poderes de cura de determinadas plantas no interior da aldeia:

Numa maloca, certas plantas são consideradas tóxicas e noutra, às vezes pouco distante, são tidas como inócuas, ou dadas com fins terapêuticos diversos. ... muito acima do valor químico e fisiológico dos remédios indígenas, estão a autoridade e a confiança que o *jorubocuro* sabe inspirar e a boa disposição moral em que se encontram os pacientes (Campos, 1913b, p.116).

Ao enfatizar a relação entre *jorubocuros* (xamãs) e seus pacientes, que se vincula às práticas de profilaxia e cura bororos, Campos procurou apontar para as características consideradas positivas no tratamento, que iam além da avaliação do suposto valor medicinal dos remédios indígenas (Vital, Hochman, 2013, p.91-92).

No artigo de Campos, destaco 24 plantas medicinais empregadas pelos bororos no tratamento de diferentes moléstias, assinalando seu nome popular e a nomenclatura indígena (ver Quadro 1). Procurei considerar apenas as plantas que foram identificadas pelo botânico Souza Britto, do Museu Nacional, que auxiliou Campos na revisão de seu artigo para capítulo de livro em 1936. Essa lista mais completa da flora das chapadas do noroeste de Mato Grosso incluía 68 plantas medicinais, 17 plantas frutíferas e 18 plantas da flora medicinal sertaneja:

**Quadro 1:** : Relação de plantas medicinais e sua aplicação entre os índios bororos, conforme Murillo de Campos

Nome popular	Sinônimos	Nome científico*	Nome indígena	Propriedades terapêuticas	Indicações etnofarmacológicas
Almecegueira	almecega, breu branco	*Protium heptaphyllum* (Aubl.) Marchand, Burseraceae		hemostática, cicatrizante, anti-inflamatória	moléstias bronco-pulmonares; confecção do *kido gúru*
Araticum	araticum-do-cerrado, araticum-do-campo	*Annona crassiflora* Mart., Annonaceae	*Ioragareu* [*Tuararagaréu*]	béquica, anti-inflamatória	tosse, dor de garganta; chá de infusão das folhas; *kido gúru* (caule carbonizado friccionado na parte anterior do peito e pescoço)
Ateira do mato	fruta-do-conde, ata, ateira	*Annona squamos*a Delile, Annonaceae	*Itchego-jorubo* [*Paragorêu iorubo*]	antidiarreico	dores de barriga; chá por infusão das folhas
Barbatimão	faveira	*Stryphnodendron barbatima*m (Vell.) Mart., Fabaceae	*Araredureu*	antirreumático	contra dores, reumatismo; chá por infusão das folhas
Bolsa de pastor	mandioquinha do campo	*Zeyheria montana* Mart., Bignoneaceae	*Baiojo* [*tugeruréu bokujíwu*]		para ter força; tiram a embira e amarram na cintura e no pescoço; contra o eczema e o impingem utiliza-se o infuso da entrecasca
Cambará	cambará do cerrado, cambará da mata	*Qualea distincha* Spreng, Vochysiaceae [?]	*Coguecureu* [*okogé ekuréu í*]	béquica	contra tosse; chá por infusão de folhas; contra bronquite
Carobinha do cerrado	caroba-do-campo	*Jacaranda rufa* Silva Manso, Bignoniaceae	*Boerubo*	antimalárico, antissifilítico	preventivo de moléstias; tratamento do impaludismo; chá por infusão das folhas
Chá de frade		*Lantana pseudothea* A.St.-Hil, Verbeneaceae[?]	*Oquájorubo* [*Oqua iorubo*]	antirreumático	resistência das pernas; bater com ramos nas pernas
Cruzeirinho	cruzeiro	*Declieuxia cordiger*a Mart. & Zucc. ex Schuet, Rubiaceae	*Boênogorubo*	cicatrizante	resfriamentos; no banho
Erva molar	erva-molar, pé-de-perdiz, alcanforeira, canela-de-perdiz, curraleira	*Croton antisyphiliticu*s Mart., Euphorbiaceae	*Boecon’epaboicagadgedo jorubo*	antirreumática, antissifilítica	dores no peito e outras pontadas; chá por decocção de folhas e raízes; fricções das folhas
Erva de passarinho		não identificado	*Jorubopagatogue*	preventivo de moléstias	usado em *kido gúru*
Faveira d’anta	fava-d’anta, faveiro-do-cerrado, faveiro	*Dimorphandra mollis* Benth., Fabaceae	*Betagaójorubo*	anti-inflamatória	moléstias epidêmicas; fricções no corpo
Fruta d’óleo	pau d’óleo, copaíba pequena	*Copaiba martii* (Hayne) Kuntze, Fabaceae	*Joruboiôcopêgaépa* [*baría í*]	moléstias dos olhos	lavagem dos olhos com o infuso das folhas
Hortelã do cerrado		*Hyptis villosa* Pohl ex Benth., Lamiaceae	*Coguridjorubo*		preventivo de moléstias; aplicação de *kido gúru*
Jatobá do campo	jatobá	*Hymenaea courbaril* L., Fabaceae	*Sumagaï*		preventivo de moléstias; aplicação de *kido gúru*
Marmelada preta	marmelada-de-cachorro	*Cordiera* sessilis (Vell.) Kuntze, Rubiaceae	*Ocoïca* [*Bóko íka*]		Evitar moléstias; *kido gúru* no peito
Paineirinha	paineirinha do campo	*Eriotheca gracilipes* (K. Schum.) A. Robyns, Malvaceae	*Aroécodudreu*		Contra pontadas; aplicação de folhas no peito
Paratudo	paratudo do cerrado	*Tecoma caraiba* Mart., Ex DC. Bignoniceae	*Boicogodureujamedoepa* [*kuógo í*]	febrífuga, tônica	amarelão, anemia pós-malárica; chá por infusão da entrecasca; emprega-se o infuso da raiz como estomático; para tratar da opilação
Pau-terra	carvãozinho	*Qualea cordata* (Mart.) Spreng, Vochysiaceae	Bareguerireu		erupções da pele; chá
Pequi	pequizeiro	*Caryocar brasiliense* Camb., Caryocaraceae	*Ecoipo* [*éko í*]	expectorante, estomático	para fabricação do *kido gúru*; carvão da madeira é comido contra a hemoptise
Piúva	ipê-roxo	*Handroanthus impetiginosus* (Mart. Ex DC) Mattos, Bignoniceae	*Emaï*		preventivo de moléstias; aplicação de *kido gúru*
Tucum		*Bactris glaucescens* Drude, Arecaceae	*Boquedaga* [*Buquedága enogo*]	antitérmica	preventivo de moléstias; *kido gúru* no peito; cozinhar o palmito e beber a água contra febres
Unha de vaca	pata-de-vaca, pata-de-boi, unha-de-boi	*Bauhinia forficata* Link., Leguminosae	*Atuboetagureu* [*Atubóe tagaréu*]	anti-inflamatória	preventivo de moléstias; fricção das folhas no corpo
Urucum	colorau, urucu	*Bixa orellana* L., Bixaceae	*nonógo*	revulsiva	

* Para uma atualização dos dados de identificação consultou-se: página eletrônica *Flora e funga do Brasil*, administrada pelo Instituto de Pesquisas Jardim Botânico do Rio de Janeiro. Disponível em: https://reflora.jbrj.gov.br/. Acesso em: 17 mar. 2023; Hartmann (1968, p, p.75-81).

Fontes: Campos (1913b, 1936); Rondon (1948); Caribé, Campos (1991).

Quase todas são referidas pelos naturalistas, que, de há muito vêm, percorrendo o interior do Brasil. Algumas, porém, não se encontram consignadas pelos mesmos, talvez por efeito da diversidade de denominações vulgares, não raro de uma significação puramente regional. Outras envolvem denominações vagas, a que se ligam várias espécies vegetais (Campos, 1936, p.13).

Determinadas plantas citadas no artigo aparecem apenas com seu nome indígena, pois é possível que o espécime coletado não tivesse sido identificado por um especialista. Segundo Hartmann (1968, p.59), boa parte da dificuldade na identificação das plantas medicinais dos bororos se origina na nomenclatura “terapêutica”, a partir da qual “encontramos as mais crassas divergências entre o testemunho dos informantes”. Nesse sentido, parece existir uma “nomenclatura comum a todo o grupo” e, ao mesmo tempo, “uma nomenclatura terapêutica quase que individual”, originada da divisão que os bororos fazem entre plantas que constituem *erúbo*, remédio, e aquelas que não apresentam essa qualidade. Isso explicaria, em parte, o comentário de Campos em relação à falta de uniformidade nos tratamentos prescritos pelos *jorubocuros*.

Na sua exposição, Campos esclarece que os curandeiros bororos utilizavam algumas plantas medicinais como recursos preventivos de doenças (*bôevéboe*). São mencionados seus usos e aplicações terapêuticos:

1º a fricção do corpo com as folhas de *atugoboétagurêu* (unha de vaca).

2º a fricção do peito com o carvão de *buoquedaga* (tucum).

3º a lavagem do corpo com o infuso de *djorubotoboépa* ou *betaódjorubo* (faveira d’anta).

4º aplicação em diversas partes do corpo do *kidoguro* de *ocoïca* (marmelada preta) ou de *coguridjorubo* (hortelã do cerrado), *sumagaï* (jatobá do campo), e e*maï* (piúva); e *ecoïpo* (pequi), *aroécodudireu* (paineirinha); *djorubopagadureu* (herva de passarinho) etc. (Campos, 1936, p.144-145).

Para o tratamento de doenças, empregam-se plantas específicas diversas, o que demonstra o conhecimento acumulado pelos bororos sobre a flora do Cerrado. Campos começa justamente tratando da malária, mais especificamente dos expedientes adotados pelos curandeiros na manifestação de sintomas como febre e calor (*magato, oôrurébôe* ou *boêru*). Em geral, o impaludismo se apresentava sob forma benigna, mas podia assumir proporções epidêmicas ao forçar a mudança das malocas, como em episódio mencionado, ocorrido no vale do rio das Mortes. Entre os recursos medicamentosos indicados para curar os acessos da doença, são mencionados o chá por infusão das folhas da carobinha do cerrado e o chá da entrecasca do paratudo, este recomendado tanto para anemia pós-malárica como para o amarelão (Campos, 1913b, p.114).

Os casos de malária eram significativos entre os bororos. Campos diagnosticou nove casos entre os 81 índios examinados (a população das cinco colônias era estimada em 1.001 indivíduos). O trânsito de tropas militares e de expedições científicas pelo destacamento militar de Sangradouro e a existência desde 1900 da Estação Telegráfica de São Lourenço e de um posto indígena recém-instalado pelo SPI podem ter colaborado, certamente, para uma maior circulação de doenças entre os bororos (Viveiros, 1958, p.122-123).

Os distúrbios reumáticos aparecem entre as queixas mais comuns. Campos (1936, p.138) chega a atribuir sua causa ao hábito dos bororos de dormir à volta do fogo sobre esteiras de buriti e utilizar como cobertor grandes lâminas da casca da figueira. Entre as opções terapêuticas indicadas pelos curandeiros para esses males, menciona o chá por infusão das folhas da erva molar ou sua fricção sobre os pontos dolorosos, a fricção das folhas da ateira do mato, a aplicação demorada no lugar dolorido das folhas da paineirinha do campo, e o chá por infusão das folhas do barbatimão (Campos, 1913b, p.114).

A mesma exposição à friagem é assinalada também como responsável pelas “moléstias gripoides”, que, juntamente com os casos de bronquite, somaram sete ocorrências. O *barí* recomenda diversos chás e fricções de plantas: chá por infusão das folhas de *cujarépa* e aspiração desta, convenientemente triturado, o chá por infusão das folhas de araticum ou a fricção do caule carbonizado no peito e no pescoço; o banho geral com a infusão de cruzeirinho; o chá das folhas de cambará da mata e a fricção com as folhas de araticum na testa (Campos, 1913b, p.114; 1936, p.146).

Definidas como “frequente e graves”, as doenças broncopulmonares teriam igual correlação com o “hábito de dormir junto ao fogo”, aparecendo principalmente durante a estação seca (Campos, 1913b, p.114). Tais afecções são tratadas com uma camada de urucum sobre o tronco e com a aplicação no peito, nas têmporas, nas regiões deltoides de *Quidoguro* ou *kido gúru*, uma mistura de resina de almecegueira e do carvão feito de algumas plantas, de “ação revulsiva e caustica” (p.114).

O elevado número de casos reportados de doenças oftálmicas (catarata, conjuntivite, estafiloma) e de cegueira levou Campos a considerá-las o verdadeiro “flagelo destes índios” (Campos, 1913b, p.114), que alcançou pelo menos 22 indivíduos, ou cerca de 1/4 do total de doentes atendidos. Se observamos o Quadro 2, apenas na aldeia do Córrego Grande não se registraram moléstias oculares, enquanto na colônia do Sagrado Coração há maior concentração de casos de conjuntivite e cegueira, além de todos os de catarata.

**Quadro 2:** : Doenças ou moléstias registradas entre os indígenas das Colônias Bororo do Leste de Mato Grosso visitadas pelo médico Murillo de Campos em 1911

Nome da doença	C1*	C2*	C3*	C4*	C5*	Total
Impaludismo (malária)	2	-	2	3	2	9
Reumatismo	2	-	-	5	1	8
Conjuntivite/cegueira	3	-	4	9	1	17
Catarata	-	-	-	5	-	5
Estafiloma	1	-	1	-	1	3
Gripe/bronquite	6	-	-	1	-	7
Disenteria	-	-	-	-	1	1
Gastroenterite	-	-	1	-	-	1
Doença gastrointestinal	-	-	-	1	-	1
Varicela	-	-	-	-	3	3
Anemia	-	-	-	2	-	2
Bócio	-	1	-	-	-	1
Escoliose	1	-	-	-	-	1
Dor ciática	1	-	1	-	-	2
Dismenorreia	-	-	-	2	-	2
Amarelão	-	-	-	1	-	1
Escrofulose	-	-	-	2	-	2
Nevralgia dentária	1	-	-	2	-	3
Infestação bicho-de-pé	-	1	-	-	2	3
Úlcera do nariz	-	-	-	-	1	1
Outros casos	2	6	-	4	-	12
**Total**	**19**	**8**	**9**	**37**	**12**	**85**

* C1 – Colônia São Lourenço; C2 – Aldeia do Córrego Grande; C3 – Colônia dos Barreiros; C4 – Colônia do Sagrado Coração; C5 – Colônia do Sangradouro.

Fonte: Campos (1913b, 1936).

Essas doenças dos olhos denominadas *iôcocóri* se iniciam como uma conjuntivite e podem evoluir, ao longo de meses e anos, por descuido e exposição constante à fumaça, para estafilomas, sinequias e cegueira. Campos (1913b, p.114) atribui esse agravamento supostamente ao costume de se arrancar os cílios, mas parece considerar a hipótese fornecida por um especialista que suspeitava tratar-se de esporotricose ocular (Campos, 1936, p.139). O tratamento indicado consistia na lavagem dos olhos com o chá por infusão das folhas de fruta d’óleo e a aplicação demorada das folhas aquecidas de *aïgejoruboboecuépa* ou de *maïa* (não identificadas) sobre as pálpebras antes de dormir.

Em julho de 1911, quando da visita do SPI, estourou uma epidemia de catapora na colônia do Sangradouro. Na verdade, surtos epidêmicos de gripe, escarlatina, roséola, febre palustre e febre amarela começaram a se tornar bastante frequentes entre os bororos no começo do século XX, em decorrência da ação dos missionários e da Comissão Rondon. A concentração de índios nas missões, fosse para se “defender de ataques de seus inimigos”, fosse para ter acesso a “assistência no caso de epidemias”, favorecia, por seu lado, o “contágio e a propagação das doenças” (Novaes, 1993, p.149). Em 1904, por exemplo, uma epidemia de gripe atingiu as colônias salesianas. Nessa ocasião, os missionários aproveitaram a ausência do *bari* para “ministrar remédios aos bororos, assegurando-lhes que em dois dias estariam curados, o que de fato ocorreu” (p.147). Outra epidemia, de febre palustre, provocou muitas mortes na missão do Sagrado Coração em 1905, e, apesar do aumento da confiança dos índios no padre Giovanni Balzola, estes mantiveram seu apreço ao *bari* Totó. Na missão de Sangradouro, em 1913, todos os 88 índios recém-chegados foram acometidos de gripe e escarlatina. As doenças atingiam também os indígenas envolvidos na construção da linha do telégrafo, como a epidemia de sarampo que irrompeu em 1901 no acampamento no contraforte Água Branca, próximo a Coxim. Rondon chegou a comentar sobre os inúmeros rituais fúnebres e das dificuldades, para os bororos, de realizá-los fora de seu ambiente (Viveiros, 1958, p.132-133).

A terapêutica profilática para as chamadas “moléstias eruptivas” se iniciava com um banho geral com o infuso das folhas de faveira d’anta, a aplicação de um *kido gúru,* preparado com os frutos de marmelada preta ou do pequi, na testa e nos deltoides, com carvão de erva de passarinho e fricção do corpo feita com as raízes de unha de vaca. Especificamente para varíola, recomendava-se o chá da entrecasca de pau-terra; já para catapora, aplicações de *kido gúru* de almacega e do carvão do fruto do jatobá (Campos, 1913b, p.114-115).

Uma ocorrência comum era a infestação por bicho-de-pé (*Tunga penetrans*), encontrada em muitos pacientes acometidos por forte erupção pruriginosa. Sua extração era feita com espinho, passando no lugar ou em todo o corpo *nônôgo* ou *nonógo*, pasta vermelha de urucum (Ribeiro, 2006, p.21; Hartmann, 1968, p.61).

Durante a cerimônia do *marído*, dança do buriti, que ocorre no centro do pátio da aldeia, dois grupos de seis homens cada um “dão a volta aos ossos do morto, carregando no alto da cabeça grande disco feito de talos verdes de buriti, de um peso médio de 9 arrobas, e batendo compassadamente com os pés sobre o chão” (Campos, 1913b, p.115). Esse enorme esforço provocava acidentes e levava muitas vezes à morte do indivíduo ou a um quadro de insuficiência cardíaca, como teve oportunidade de constatar na colônia São Lourenço. Assim, para evitar fadiga muscular, recomendava-se amarrar embira com folhas de bolsa de pastor ao nível dos tornozelos e friccionar os membros com folhas de chá de frade.

Apesar do trânsito nas cercanias das colônias, Campos registrou apenas um caso de bócio, mencionando ainda a ausência de casos de doenças venéreas, beribéri, lepra, tuberculose e alcoolismo entre os índios, males que assolavam todas as localidades que visitara. Por fim, nem sequer encontrou vestígio de barbeiros nas malocas.

## Considerações finais

Ao publicar seu relatório médico no formato de um denso artigo científico, o capitão Murillo de Campos teve a oportunidade de explorar com mais profundidade que num relatório burocrático informações sobre as práticas de cura e conhecimentos ancestrais sobre plantas medicinais entre os índios bororos do leste de Mato Grosso.

O aspecto original do trabalho se encontra justamente na sua abordagem revelada no cuidado em tentar explicar alguns aspectos das práticas de cura dos bororos. Para isso, contou com as informações fornecidas pelos *jorubocuros* e por outros índios bororos com os quais entrou em contato durante sua passagem nas colônias, embora não haja registro ou menção específica a algum desses informantes em particular. A preocupação que Murillo de Campos tem em descrever as propriedades terapêuticas e indicações etnofarmacológicas das plantas medicinais empregadas pelos xamãs, assinalando seu nome indígena e popular, assim como algumas especificidades dos tratamentos, demonstra percepção quanto à relevância daqueles registros e das amostras que poderiam ser utilizadas por cientistas em pesquisas laboratoriais.

Conforme assinalado, a busca dos médicos militares por maior autonomia, especificamente na CLTEMTA, permitiu que pudessem atuar mais ativamente nas expedições de reconhecimento, ao auxiliarem os cientistas de diferentes instituições ou efetuarem suas próprias pesquisas de campo e investigações preliminares nas comunidades e aldeamentos indígenas visitados, o que redundou em produção etnográfica e científica publicada pela Comissão Rondon. Trata-se de um conhecimento produzido “em trânsito”, que surgiu da interação entre comunidades heterogêneas de especialistas (médico e *jorubocuros*) em zonas de circulação interculturais, que promoviam transformações e reconfigurações das diferentes práticas e conhecimentos.

## Data Availability

Não estão em repositório.

## NOTAS

1 Vinculada à Faculdade de Medicina do Rio de Janeiro, foi criada em 1887, como uma revista semanal de divulgação de uma medicina nacional. No início do século XX, o tema do combate às epidemias e as campanhas de saneamento levaram a uma mudança no perfil da revista em favor do médico pesquisador dedicado à tarefa de buscar a cura das moléstias (Schwarcz, 1993, p.286-287, 293-294).

2 O Serviço de Proteção aos Índios e Localização de Trabalhadores Nacionais passou a funcionar em 1910, sob a direção de Cândido Rondon, que o concebeu como desdobramento de ações da CLTEMTA, para sedentarizar e nuclear índios a fim de colaborar no processo de formação do trabalhador nacional (Maciel, 1998, p.171).

3 Trata-se de povo indígena conhecido como *otuques, bororós* ou *boe* (autodenominação), que ainda habita o leste desse mesmo estado. Falam a língua bororo, autodenominada *boe wádaru*, que pode pertencer ao tronco linguístico macro-jê (Urquiza, 2007).
