# Supplementary material for: Saberes médicos e práticas de cura tradicionais dos bororos: relatos de uma expedição ao sertão de Mato Grosso, 1911
Source: Hist Cienc Saude Manguinhos. 2025 Jan 10;31:e2024073. [Article in Portuguese] doi: 10.1590/S0104-59702024000100073 (PMC11723583; doi:10.1590/S0104-59702024000100073)
Supplement: Translation

# Translation: Medical knowledge and traditional healing practices of the Bororo: reports from an expedition to the interior of Mato Grosso, Brazil, 1911

**DOI:** 10.1590/S0104-59702024000100073en

**Authors:** Robson Mendonça Pereira

**Affiliations:** i Professor, Universidade Estadual de Goiás. Anápolis – GO – Brazil. robsonmenper@hotmail.com

**Keywords:** Medicinal plants, Rondon Commission, Intermediary, Murillo de Campos (1887-1968)

## Abstract

This text analyzes the medical reports of doctor Murillo de Campos on an expedition by the Rondon Commission to the Brazilian state of Mato Grosso in 1911. Originally published as a scientific article, the report begins by detailing the nosological profile in the places visited and the customs of the inhabitants. It then presents a detailed fieldwork report into the healing practices of the Bororo indigenous people in eastern Mato Grosso. An investigative approach is employed to describe the therapeutic methods used by the healers, particularly the extensive use of medicinal plants. The analysis demonstrates that Campos acted as an intermediary between medical knowledge and Bororo healing practices.

## Participation of a military doctor in the Rondon Commission

On March 22, 1913, *Brasil-Médico*, an important weekly journal in the areas of public health and tropical medicine,^1^ published in its Original Works section an article entitled “Notas do interior do Brasil: do Rio de Janeiro a Cuiabá (via Goiás)” (Notes from the interior of Brazil: from Rio de Janeiro to Cuiabá [via Goiás]), by Murillo de Souza Campos (1913b, p.111-116). Campos was a physician who had been working in the sanitary service of what has come to be known as the Rondon Commission, whose official name was the Commission for Strategic Telegraph Lines from Mato Grosso to Amazonas (Comissão de Linhas Telegráficas Estratégicas do Mato Grosso ao Amazonas, CLTEMTA). In his article, he presents a detailed scientific and medical account of the long journey he undertook with an inspection group from the recently created Service for the Protection of Indians (Serviço de Proteção aos Índios, SPI),^2^ whose final destination had been Cuiabá, in Mato Grosso state, where the commission had its operational headquarters.

Although Campos remained with the CLTEMTA for just a short while, this immersive experience gave rise to a wealth of material, as evidenced in this article and others, which was subsequently published together in *Interior do Brasil: notas médicas e etnográficas* (Interior of Brazil: medical and ethnographic notes) (Campos, 1936).

Murillo de Souza Campos’s trajectory can be seen as similar to that of others who served with the CLTEMTA. He was born in 1887 in Amparo, a small town in São Paulo state. In 1908 he graduated from the Rio de Janeiro Faculty of Medicine, and in the following year he joined the army’s health service. In May 1910, he embarked as a first lieutenant for Cuiabá and then to the CLTEMTA advanced camp on the banks of the Juruena River to carry out his first mission.

When Campos took up his post with the CLTEMTA, it had three clinicians: Joaquim Augusto Tanajura, João Florentino Meira de Faria, and Paulo Fernandes dos Santos. Their work took them to the different camps along the route of the new telegraph line, which involved traveling great distances to care for the many workers who were injured or sick, usually from malaria or other endemic tropical diseases. However, military doctors were also deployed to serve on the commission’s reconnaissance expeditions.

At that time, the CLTEMTA health service was being reorganized, giving its doctors a wider scope of action, in line with changes being made in the army health service (Vital, 2011, p.38-40). One key tenet of this process was to ensure that the doctors had adequate authority and autonomy.

The head of the CLTEMTA tasked Joaquim Tanajura with drawing up the instructions for the new health service, which was approved on May 22, 1910 (CLTEMTA, [1911], p.109-113). Two key decisions were immediately made: to install infirmaries in Santo Antônio do Madeira and Serra do Norte and to create a malaria prophylaxis service, which was urgently needed to prevent new epidemics of the kind that paralyzed the construction work between July 1910 and July 1911 (Diacon, 2006, p.81; Vital, 2011, p.69-71).

The doctors working at the infirmaries were to have autonomy to take decisions on such matters as the isolation of patients and prescription of daily doses of quinine to soldiers and civilians. Furthermore, the health service’s remit was expanded by giving the doctors the additional “functions of researchers and analysts of the region’s diseases, advisors to the heads of expeditions, and responsibility for malaria prophylaxis and care for the sick and injured” (Caser, Sá, 2010, p.372-373).

Effectively, doctors were being encouraged to act like scientists, insofar as they were permitted, “for experimental purposes, to test the cultivation of recommended plants in swamplands and vegetables for the workers’ consumption whenever possible, despite not being able to reap any immediate benefits.” They should also write up detailed observations on cases of ulcers, myiasis, beriberi, and “any disease that [they] deem worthy of analysis, particularly studying their causes and the insect vectors that may be responsible for their transmission” (CLTEMTA, [1911], p.111).

One indication that the CLTEMTA doctors were preparing to work as scientists was that many underwent some type of training in how to investigate the main tropical diseases in the northwest region. For instance, the medical captain José Antônio Cajazeira, who served on the Roosevelt-Rondon Scientific Expedition (1913-1914), completed a full six-month course at the Oswaldo Cruz Institute (Instituto Oswaldo Cruz, IOC). He was then allowed to stay a further five months at the institute to train in other subjects (CLTEMTA, 1916, p.4). In his report, he argued that doctors should produce their own knowledge on tropical diseases. It was “indispensable” to have a “small, portable microscope, with its box of essential reagents, chosen and prepared by the expedition doctor, who, in addition to being skilled in handling such a device, must know more or less the diseases prevalent in the area to be explored in order better to organize his mobile clinic” (p.14).

The doctors counted on traditional knowledge they obtained from local people to “undertake studies on diseases and their cycles in the northwest of the country” (Vital, Hochman, 2013, p.78) and to investigate the specific characteristics of the diseases that plagued the workers, such as malaria, ancylostomiasis (hookworm infection), and beriberi. Generally, the only people they could consult were indigenous and *sertanejos* (backcountry settlers). They supplemented this knowledge with observations about “river flood cycles and migratory flows” (p.78) to build up an epidemiological profile, from which they would then “devise strategies, based on tropical medicine, in order to prevent serious epidemics among the Commission workers” (p.78-79).

To develop a better understanding of how these doctors acquired knowledge about “unfamiliar events, places and people” (Latour, 2000, p.362),^3^ I draw on Bruno Latour’s understanding that this cognitive process is only possible “by considering a whole cycle of accumulation” (p.357) of knowledge. As observers working from a distance, these doctors used the same methods as had been developed by sanitarians and epidemiologists to carry out their fieldwork in far-flung parts of the country.

On his own reconnaissance expeditions, Rondon was also accompanied by scientists. Often, the doctor assigned to take care of the sanitary service was involved, together with some officers, in collecting plants, insects, and animals, which gave them a practical grounding in how to handle such specimens.

In July 1910, Campos embarked on his first mission as a member of a reconnaissance expedition to study the plant life of the Juruena River. Organized by Rondon on the request of the botanist Frederico Carlos Hoehne, the expedition was led by Captain Manuel Teóphilo da Costa Pinheiro. While on the expedition, Campos collected zoological, ethnographic, and plant matter as part of a project run by Hoehne, for the National Museum of Rio de Janeiro (Museu Nacional do Rio de Janeiro) (Sá, Sá, Lima, 2008, p.794).

Campos (1913a) published the findings of his investigative work in “Notes from the interior of Brazil: northwest of Mato Grosso,” which he published in the journal *Arquivos Brasileiros de Medicina*. The text presents the medicinal plants used by the *sertanejo* and indigenous populations in the region, as well as a survey of the ailments of the Parec and Nambikwara peoples and their respective healing practices. Parallels can be seen between this article and another of the same name in *Brasil-Médico*. In both, Campos acknowledges the work of several collaborators, including local people (rubber tappers, indigenous people etc.), who provided him with important information about the most prevalent diseases in the places he visited and the traditional knowledge and healing practices.

In this sense, Campos was like other doctors from the commission, who were capable of acting as a kind of intermediary (in the sense given by Raj, 2007), insofar as they could perform some degree of mediation, whether as an interpreter or translator, at the point of contact between two worlds. In Campos’s case, the two worlds in question were those of scientific medicine and traditional indigenous knowledge (Antunes, 2019, p.43).

The commission’s doctors thus constituted a group of specialized intermediaries who had the means to evaluate practical knowledge. This knowledge inevitably depended on negotiations between specialists and local collaborators for it to be produced and certified (Raj, 2007, p.14). It was, in the terms of Callon, Lascoumes, and Barthe (2009), the result of “open-air” research (*recherche de plein air*), as distinct from the kind of knowledge produced by the field sciences or in laboratories.

### Accounts of an inspection expedition on the service of the SPI

Campos’s journey to the CLTEMTA camp began in May 1911, when he took a train on the Central do Brasil railway from Rio de Janeiro to São Paulo. From there, he journeyed inland to the village of Bauru, where the Noroeste do Brasil railway began, whose route took it across a huge swathe of São Paulo state where predatory land-grabbing was rife. The northwest region was being mapped at the same time by the São Paulo Geographic and Geological Commission, and was represented on maps as if it were a vast, empty, unknown space. This strategy provided the conditions for land grabbers to occupy and claim ownership of areas of land, then negotiate their sale to coffee growers (Arruda, 2000, p.123-124; Dean, 1996, p.229-230). As a result, new conflicts broke out between the white settlers and indigenous people, resulting in their murder or displacement to official settlements (Mota, Schraiber, 2021, p.440-445).

Campos (1913b, p.111) describes how the landscape and environmental conditions favored the reproduction of the *Anopheles* mosquito, the vector of malaria. Along the longest section of the railway, it was common for the workers to be infected, and many fell ill and died.

The impairment of the lives of workers by the most prevalent endemic diseases in the northwest of São Paulo was remarked upon by Arthur Neiva, an IOC researcher hired by the railway company to “fight malaria on the construction sites” (Benchimol, Jogas Junior, 2020, p.45). Neiva felt that the exploitation of the workers, the invasion of indigenous lands, and the incursion into the forests had been instrumental in the “rapid and profound spread” of leishmaniasis. Known in the region as “Bauru ulcer,” it had first been diagnosed in Latin America in 1909 by researchers from Institut Pasteur and the São Paulo Bacteriological Institute (Instituto Bacteriológico Paulista), at a time when there was intense international debate on the topic (Mota, Schraiber, 2021, p.445-446; Benchimol, Jogas Junior, 2020, p.13).

When he alighted at Itapura station, the final stop of the railway, near the mouth of the Tietê River, Campos carried out tests on soldiers from the Caçadores battalion and members of the Kaingang people who lived close to an SPI post. In addition to malaria, he identified cases of other endemic diseases, such as ancylostomiasis, goiter, syphilis, and alcoholism. Remarking on what remained of the Kaingang, he noted, albeit *en passant*, the miserable conditions in which they lived. They had resisted the encroachment of the railway into their territory, but had been decimated and had their villages destroyed by Indian-killers hired by the company and squatters (Ghirardello, 2002, p.42-43).

Campos also remarks on the absence of beriberi, reported by the doctors who had attended the construction of the São Paulo section of the railway (1905-1910), even though it was common in the northwest of Mato Grosso, which he attributed to differences in the quality of the workers’ diets (Ghirardello, 2002, p.40, 44). Curiously, no cases of yellow fever or leishmaniasis were reported either, the latter being particularly remarkable since its incidence remained high in the northwest zone throughout the 1910s. These diseases were considered important in studies of the epidemiological profile of the Central-West and Amazon regions undertaken by the IOC (Ghirardello, 2002, p.41; Lima, 2013, p.137-138; Benchimol, Jogas Junior, 2020, p.44-45).

When Campos was in Uberaba, on his way across Triângulo Mineiro (western Minas Gerais), he noticed that the illnesses of the local people were similar to those found in the northwest of São Paulo state. He mentions leprosy and also briefly recounts local information gathered on two skin diseases: “wildfire” (pemphigus foliaceus), of unknown etiology, and *caphobira* (scabies), “which appears to be linked to syphilis” (Campos, 1913b, p.111). He comments that he found houses infested with triatomine bugs on a farm near the village of Araguari. This mention is significant, as extensive searches for triatomines were being commenced in the interior of the country at the time. The specimens were sent for examination at the laboratories of institutes like IOC in order to ascertain whether they were carrying *Trypanosoma cruzi*, which causes Chagas disease (Brazilian trypanosomiasis) (Silva, 2021, p.124-125). Vital and Hochman (2013, p.89) suggest that Campos may have agreed with Carlos Chagas’s hypothesis that the goiter that was endemic in rural areas of Brazil was a “clinical manifestation of trypanosomiasis,” and thus different from the goiter among the Europeans.

After crossing the Paranaíba river, Campos went to several parts of Goiás state. In his assessment of the habits of the *sertanejo* settlers, which is far more damning, he describes a population living in a permanent state of insalubrity fostered by neglect on the part of the authorities:

The people of Goiás, isolated from the rest of the nation, habitual and uneducated, live scattered along the highways, forming a few villages. In general, their housing is poorly finished and unhygienic. Situated on the outskirts of the forest in a small clearing, it is always close to the water. It is usually accompanied by a barn, a pigsty, a chicken coop, and nearby a paddock. As a result of this unhygienic situation, at nightfall, culicids fly into the houses and, I have been told, triatomine bugs, jiggers, and ticks are commonly found (Campos, 1913b, p.111).

It is important to note that it was during the 1910s and 1920s, under Brazil’s “rural sanitation campaign,” that a growing consensus began to be voiced by the medical community regarding the need for state actions to combat the country’s various endemic diseases. This was designed to improve the health of the *sertanejo* people in the interior of the country and incorporate them into the budding national community. A diagnosis of the social ills was proposed, along with a set of centralized actions to be taken by the federal government, all with the aim of intervening in rural areas. These measures were no longer influenced by the concepts of racial and climatic determinism, which had influenced the first phase of health reforms (Hochman, 1998, p.91-93).

According to Vieira (2022), doctors from the CLTEMTA, like Campos himself, had unparalleled means to carry out clinical studies of rural endemics. Even though they were working in areas that were deemed peripheral from the perspective of national science, their research provided valuable contributions to the “scientific network” of major institutions (like the IOC), where the same diseases were studied (Vieira, 2022, p.17-18; Schweickardt, 2009, p.26-28). This knowledge produced “in transit” arose from interactions between heterogeneous communities of specialists in spaces of intercultural encounter, which fostered transformations and reconfigurations of different practices and knowledge (Raj, 2007; Silva, 2021; Gesteira, 2022, p.41, 55).

Some CLTEMTA doctors made detailed descriptions of “the customs, perceptions, and knowledge” that were common among the *sertanejos* they came across (Vital, Hochman, 2013, p.86). Some criticized the way they “dealt with illnesses” (p.86), elaborating on the innocuous nature of the healing practices of the different kinds of healers and practitioners, while others avoided passing judgment and pointed to the “need for State action in the education of the inhabitants” (Vital, Hochman, 2013, p.86).

Campos’s observations and comments oscillated between these two positions. One excerpt from his report demonstrates the former: a quite disparaging appraisal of the people he came across in the states of Goiás and Mato Grosso, whom he understood as “empty repositories associated with ignorance, disease, and the archaic” (Silva, 2021, p.197), all of which could be tackled by medical practitioners (Campos, 1913b, p.112).

As for the latter position, Campos demonstrates interest in how certain ailments were understood locally. One example of this concerns the curious explanation given by the people of Goiás about the “water origin” of goiter, according to which certain sources were responsible for producing it, while others could alleviate it. At the time, the etiology of goiter was unknown, although some medical hypotheses related it to “poor water quality, heredity, and a lack of iodine in the human organism” (Silva, 2021, p.11). Campos points out that symptoms such as anemia and intermittent fever – common to different endemic diseases – raised doubts among the doctors, who would even end up resorting to popular knowledge and the remedies produced domestically and by healers using medicinal plants from the Cerrado.

After crossing the Araguaia River, the expedition continued towards the east of Mato Grosso. Campos’s first impression of that region was that it was less inhabited than the south of Goiás. However, it exhibited the same “customs and the same lack of individual and domestic hygiene” (Campos, 1913b, p.112). The diseases he highlighted among the countless others presented by the patients he treated were goiter, malaria, ancylostomiasis, leprosy, syphilis, gonorrhea, and beriberi.

Campos regarded beriberi as a more serious problem than malaria when judging the impact it had on the “men he examined, which included Pareci and Nambiquara Indians, rubber tappers, and CLTEMTA workers” (Caser, Sá, 2011, p.495). He hypothesized that the incidence of beriberi in the northwest region was related to deficient agricultural production, which made it difficult to supply food to the military garrisons and scientific and engineering commissions, who imported food from Rio Grande do Sul or Argentina. Given the way it was stored and the distances it was transported, this food rarely arrived in a “good state of conservation” at the camps (Campos, 1936, p.73). This, Campos felt, was why the disease was so common both endemically, as was seen in the garrisons of Coimbra and Corumbá Fort, and epidemically, as witnessed during the building of the Noroeste do Brasil railway (1909) and in the work of the CLTEMTA (1910) (Campos, 1913b, p.112-113).

In fact, between April and June 1910, Campos witnessed an epidemic of beriberi that affected almost half of the commission’s workforce in the Juruena camp. He dedicated a considerable part of the article “Noroeste de Mato Grosso” to a detailed assessment of the patients, putting forward several hypotheses to explain the factors that were at play in making that illness so common and serious (Campos, 1913b, 1936).

In his assessment of the sanitary conditions, which he deemed subpar, he put more weight on personal hygiene than on the insalubrious environmental conditions, calling for interventions in the social conditions:

The overwhelming impression one has of *sertanejo* people is that their ailments come from ignorance and negligence rather than from the much-vaunted insalubrity of the climate. The creation of a rural hygiene service with the aim of replicating certain truths of individual and domestic hygiene, rather than the appropriate modification of the environment, a fine utopia in our time, would be a highly humanitarian measure (Campos, 1913b, p.113).

Campos’s position is thus largely aligned with the one taken by Belisário Penna and Arthur Neiva in a report on the IOC scientific expedition of 1912, when they visited several places in the Northeast and part of Goiás (Vital, Hochman, 2013, p.89). In the “portrait” they paint of the country, they hold that what is blocking progress is not so much the climate or the race of the people, but disease (Lima, 2013, p.139-140). Bacteriology research and studies of the pathologies of tropical diseases should therefore be complemented by sanitation projects, which would get people to change their ways, guide their “dietary and hygiene customs,” curb any deviant behaviors, and prevent “degeneration,” ensuring adequate sexual practices (Schwarcz, 1993, p.297).

### The Bororo Indian colonies among different ideological projects

There was a change in Campos’s discourse when he made a visit to five Bororo colonies.^4^ Located in the east of Mato Grosso state, these consisted of two autonomous colonies (São Lourenço and Córrego Grande) and three run by Salesian missionaries (Barreiros, Rio dos Tachos, and Sangradouro). The accounts and impressions of the Bororo take up at least half of the article, indicating how important Campos felt this contact was. He discusses their eating habits and customs, their beliefs about illness, the healing methods their healers used, and how they prevented ill health.

Campos’s challenge seems to lie in understanding the distinctions and characteristics of indigenous nosology in Mato Grosso – a study he had begun in an earlier investigation of the Pareci and Nambikwara peoples (Campos, 1936, p.33-58). At the same time, he contributed to the development of knowledge by conjecturing on the likely medicinal properties contained in the medicinal plants from the Cerrado (Vital, Hochman, 2013, p.93). Studies of this kind undertaken by the commission’s doctors complemented the medical and scientific efforts being made in the nascent field of Brazilian tropical medicine (Lima, 2013, p.131-141).

Campos’s narrative begins with a description of the Bororo as “a numerous tribe, partly civilized and partly still in a savage state” (Campos, 1913b, p.113). This distinction, in addition to its ethnocentric bias, is linked to Rondon’s views on contact with indigenous peoples, for whom there was a “positivist roadmap for the progress of the nation” (Tacca, 2001, p.26). In this sense, Campos’s article tows the official commission line by avoiding any mention of the Salesian missionaries, who had maintained close contact with the Bororo since the first colonies had been set up in the early 1900s (Tacca, 2001, p.26-27).

The Salesians, who reached Brazil in 1883, took the decision to set up missions in Mato Grosso in 1894, when they undertook an expedition under the leadership of Father Lassagna. The state governor, Manoel Murtinho, gave them a colony of Bororo Indians (formerly a military colony called Teresa Cristina). However, the Salesians were subsequently evicted from the colony because “in three years they had done nothing” (Novaes, 1993, p.151).

In December 1901, the Colony of the Sacred Heart of Jesus (now Meruri) was created in a place called Barreiros, close to Ribeirão dos Tachos (eighty leagues from Cuiabá), by 14 missionaries. The Bororo only appeared eight months later, in August 1902, when they built a new village, permitting the settlement of 140 Indians there (Novaes, 1993, p.152-153). The settlement grew in 1906 when the São José colony was expanded along the banks of the Sangradouro river with an area of some twenty thousand hectares to house 500 head of cattle, 100 mules, 50 sheep, and one masonry house. The number of Bororo grew steadily over the years: in 1908, there were 67 individuals, by 1910 there were 300, and in 1911 there were 374 (p.155).

Interestingly, Cândido Rondon, head of CLTEMTA, had known the Bororo since before he joined the telegraph commissions. Born in 1865 in Mimoso, Mato Grosso, he had Bororo and Terena ancestry on his mother’s aide. He had interacted with these peoples his since childhood (Viveiros, 1958, p.17-18), and this had allowed him to learn their language and customs and had influenced him in his military career. In the early 1890s, he had joined the Commission for the Construction of Telegraph Lines from Cuiabá to Araguaia, when they built a telegraph station close to Bororo settlements in the São Lourenço river valley.

In 1901, when Rondon was appointed head of the telegraph commission in Mato Grosso, he had to deal with shortages of materials and a diminished workforce due to malaria and constant desertions. He convinced the Bororo chief (*boé e-iméjera*), Oarine Ecurue, and the shaman Barú, from São Lourenço, to provide a group of 150 of their people to clear the trail for a connecting line between Coxim and São Lourenço – a task made all the more painful by the need to remove the trunks of felled trees. This work brought the Bororo under the military discipline imposed in the camp (Viveiros, 1958, p.130-131; Tacca, 2001, p.24). Rondon sought to demarcate lands and formally charged farmers who attacked the Bororo, even though this ran counter to the orders of the Mato Grosso state government, “which had created state colonization legislation that left indigenous lands at the mercy of speculators” (Urquiza, 2007, p.73).

This example gives a window on the logic that underpinned relations between CLTEMTA and the various indigenous peoples along the line. Positivists like Teixeira Mendes and Cândido Rondon criticized the actions of the religious missions, feeling that forced conversion to Christian values violated the laws of evolution. They developed a program to “govern relations with indigenous peoples” (Diacon, 2006, p.122) that was based on the strategy of protecting and assimilating them through pacification.

In June 1911, Rondon inspected the indigenous colonies run by Salesian priests on the Garças and Barreiro rivers (CLTEMTA, 1915, p.16). While there, he attended a Bororo funeral ritual (*ítaga*) and announced his intention to found an indigenous settlement on the site of the old colony of São Lourenço, with the idea of converting it into an agricultural center (Viveiros, 1958, p.342-343). This was part of an SPI strategy to transform indigenous people into national workers.

The Salesians, for their part, had been trying somewhat unsuccessfully to assimilate the Bororo through agricultural work and distance them from their old customs. Lévi-Strauss (1996) comments that when the missionaries realized how important the traditional circular layout of the Bororo villages was to their social and religious system, they convinced them to live in a village “where the houses are arranged in parallel rows” (p.233). Furthermore, the architectural structure of the huts was changed and the men’s house (*bái mána gejéwu*), a place for public ceremonies, was eliminated and a cross was erected in its place (p.232-233).

Campos (1913b, p.113) comments succinctly on the Bororo customs and some aspects of their material culture. He begins by discussing their preference for building villages close to water courses (unlike the Nambiquara and Pareci, who dwelled on the plateau) and the fact that their longhouses were large, well-built, and housed three to four families on average. These huts were not very clean inside, in contrast to the village courtyard (*bororó*). The circular format of the Bororo village was “the ideal model for representing their society and their universe” (Novaes, 1986, p.77), enabling a diametral line to divide the population into two groups: the *eceráe*, to the north, and the *tugarége*, to the south. The villages were organized into clans, “family groups that considered themselves kin by women, based on a common ancestor of a mythological nature” (Ribeiro, 2006, p.29). Featured in the middle of the village courtyard was the men’s house (*bái mána gejéwu*), a rectangular hut that served as a temporary residence for married and single men and a place reserved for the clan chiefs’ ceremonies (Ribeiro, 2006, p.30).

As for their eating habits, they hunted and fished and also kept a few corn and cassava crops. They supplemented their diet with fruit from the Cerrado, like palm heart, coconut, pinuela, honey, and fermented juice of *acory* and buriti.

### Bororo healing practices

When Campos embarks on his descriptions of Bororo healing practices, he points out that it is a “caste privilege.” There are, however, some “*jorubocuro* doctors who practice it in virtue of their intelligence and observation” (Campos, 1913b, p.114). A *jorubocuro*, or *jorubo cure*, was a type of healer whose father acquaints them with the “secret of illnesses and knowledge of remedies” (p.114) from their earliest years. A *jorubocuro* directs their powers towards both disease prevention (prophylaxis) and treatment, which may be specific or a panacea. As for the notion of disease (*djorubo*), this is “nothing more than its most obvious symptoms: pain, fever, cough, vomiting etc.” (Campos, 1936, p.144), whose cause is supernatural and of external influence. According to Hartmann (1968, p.27, 30), the term *jorubo cure* designates a remedy or substance from a plant, its appositive being *erúbo* and its variants, *orúbo* and *jorúbo*. It is frequently found in the Bororo botanical nomenclature and refers to “those plants used in prophylactic and curative medicine” (Hartmann, 1968, p.30).

Evidently, there are some inaccuracies in Campos’s description of Bororo healing practices, since he had to limit his investigation to the illnesses presented by the indigenous patients he treated in the colonies he visited. Still, he had time to gather accurate information about the healers’ customs and procedures within the limited ethnological knowledge of the time.

Viertler (1987) explains that there are three types of Bororo healers or shamans who are responsible for the integrity of the community: the *bari*, shaman of spirits; the *aroe etawara are*, shaman of souls; and the *erubudobu epa*, lord of remedies. The first two, whose powers come from “extraordinary individual contact with the supernatural” (p.207-208), have the function attributed to them as a “matter of office, and not of clan or birth” (p.207). In Bororo cosmogony, the *bari* and the *aroe etawara are* are associated with two distinct forces: the former, to the *bope* (“spirits from nature associated with certain plants and animals”), and the latter, to the *aroe* (“water spirits and souls of the dead”). They represent a kind of dualism that refers to a mythical past in which the ancient population of farmers was understood to have merged with a more recent population of hunter-warriors. Meanwhile, the *erubudobu epa* is associated with the *erubo-mage*, which are “magical forces associated with certain plants from field and forest used as medicines or poisons” (Viertler, 1987, p.207).

The *bari* and *aroe etawara are* heal people by “extracting foreign bodies by suction” (Albisetti, Venturelli, 1962, p.115). They extract these foreign bodies, responsible for the disease, by the “‘ingestion of smoke’ from the cigars they are always offered, together with strong salivation” (p.239; emphasis in original). For his part, the *erubudobu epa* (“lord of remedies”) acts in secret, ingesting or physically or symbolically approaching the patient with some substance of plant origin, and may also cast spells against enemies.

This last type of Bororo healer or shaman could well be the *jorubocuro* doctor to whom Campos refers, as he is a valuable informant who has ancestral knowledge about the medicinal plants from the Cerrado and how to use them. Treatment and healing practices varied depending on the opinion of each shaman and the supposed healing powers of certain plants inside the village:

In one longhouse, certain plants are considered toxic and in another, sometimes not far away, they are considered harmless or given for different therapeutic purposes. ... far above the chemical and physiological value of indigenous remedies, is the authority and confidence that the *jorubocuro* is able to inspire and the good moral disposition of the patients (Campos, 1913b, p.116).

By emphasizing the relationship between *jorubocuros* (shamans) and their patients, which is linked to Bororo prophylactic and healing practices, Campos sought to point to the positive aspects of such practices, which went beyond judging the supposed medicinal value of the indigenous remedies (Vital, Hochman, 2013, p.91-92).

In Table 1, I detail 24 medicinal plants Campos identifies as being used by the Bororo to treat different ailments, providing their popular and indigenous names. This list includes only those plants that were identified by the botanist Souza Britto, from the National Museum, who helped Campos revise his article for a book chapter in 1936. This more complete list of the flora of the plateaus in the northwest of Mato Grosso includes 68 medicinal plants, 17 fruiting plants, and 18 medicinal plants from the region:

**Table 1:** : Medicinal plants and how they were used by the Bororo, according to Murillo de Campos

Common Name	Synonym	Scientific Name*	Indigenous Name	Therapeutic Properties	Ethnopharmacological Indications
Almecegueira	almecega, breu branco	*Protium heptaphyllum* (Aubl.) Marchand, Burseraceae		hemostatic, wound healing, anti-inflammatory	broncho-pulmonary diseases; making the *kido gúru*
Araticum	araticum-do-cerrado, araticum-do-campo	*Annona crassiflor*a Mart., Annonaceae	*Ioragareu* [*Tuararagaréu*]	antitussive, anti-inflammatory	cough, sore throat; infusion of leaves; *kido gúru* (charred stems rubbed on the back of the chest and neck)
Ateira do mato	fruta-do-conde, ata, ateira, sugar apple	*Annona squamos*a Delile, Annonaceae	*Itchego-jorubo* [*Paragorêu iorubo*]	antidiarrheal	stomach ache; infusion of leaves
Barbatimão	faveira	*Stryphnodendron barbatima*m (Vell.) Mart., Fabaceae	*Araredureu*	anti-rheumatic	for pain, rheumatism; infusion of leaves
Bolsa de pastor	mandioquinha do campo	*Zeyheria montana* Mart., Bignoneaceae	*Baiojo* [*tugeruréu bokujíwu*]		to have strength; fiber removed and tied round the waist and neck; infusion of inner bark for eczema and dermatitis
Cambará	cambará do cerrado, cambará da mata	*Qualea distincha* Spreng, Vochysiaceae [?]	*Coguecureu* [*okogé ekuréu í*]	antitussive	antitussive; infusion of leaves; for bronchitis
Carobinha do cerrado	caroba-do-campo	*Jacaranda rufa* Silva Manso, Bignoniaceae	*Boerubo*	anti-malarial, anti-syphilitic	preventive remedy; for malaria; infusion of leaves
Chá de frade		*Lantana pseudothea* A.St.-Hil, Verbeneaceae[?]	*Oquájorubo* [*Oqua iorubo*]	anti-rheumatic	to strengthen legs; hit legs with branches
Cruzeirinho	cruzeiro	*Declieuxia cordige*ra Mart. & Zucc. ex Schuet, Rubiaceae	*Boênogorubo*	wound healing	colds; in baths
Erva molar	erva-molar, pé-de-perdiz, alcanforeira, canela-de-perdiz, curraleira	*Croton antisyphiliticu*s Mart., Euphorbiaceae	*Boecon’epaboicagadgedo jorubo*	anti-rheumatic, anti-syphilitic	chest pain and other intermittent, acute pain; infusion of leaves and roots; rubbing with leaves
Erva de passarinho		not identified	*Jorubopagatogue*	preventive remedy	used in *kido gúru*
Faveira d’anta	fava-d’anta, faveiro-do-cerrado, faveiro	*Dimorphandra mollis* Benth., Fabaceae	*Betagaójorubo*	anti-inflammatory	epidemic diseases; rubbed on the body
Fruta d’óleo	pau d’óleo, copaíba pequena (copaiba)	*Copaiba martii* (Hayne) Kuntze, Fabaceae	*Joruboiôcopêgaépa* [*baría í*]	eye conditions	rinse eyes with infusion of leaves
Hortelã do cerrado		*Hyptis villosa* Pohl ex Benth., Lamiaceae	*Coguridjorubo*		preventive remedy; use as *kido gúru*
Jatobá do campo	jatobá	*Hymenaea courbaril* L., Fabaceae	*Sumagaï*		preventive remedy; use as *kido gúru*
Marmelada preta	marmelada-de-cachorro	*Cordiera* sessilis (Vell.) Kuntze, Rubiaceae	*Ocoïca* [*Bóko íka*]		disease prevention; *kido gúru* on the chest
Paineirinha	paineirinha do campo	*Eriotheca gracilipes* (K. Schum.) A. Robyns, Malvaceae	*Aroécodudreu*		for intermittent, acute pain; leaves applied to the chest
Paratudo	paratudo do cerrado	*Tecoma caraiba* Mart., Ex DC. Bignoniceae	*Boicogodureujamedoepa* [*kuógo í*]	antipyretic, tonic	intestinal hookworm disease, post-malarial anemia; ingestion of infusion of inner bark; infusion of the root for buccal conditions; for hookworm disease
Pau-terra	carvãozinho	*Qualea cordata* (Mart.) Spreng, Vochysiaceae	Bareguerireu		rashes; tea
Pequi	Pequizeiro, souari	*Caryocar brasiliense* Camb., Caryocaraceae	*Ecoipo* [*éko í*]	expectorant, mouth disorders	used in *kido gúru*; ingestion of charcoal made from the wood to treat hemoptysis
Piúva	ipê-roxo (purple ipe)	*Handroanthus impetiginosus* (Mart. Ex DC) Mattos, Bignoniceae	*Emaï*		preventive remedy; use as *kido gúru*
Tucum		*Bactris glaucescens* Drude, Arecaceae	*Boquedaga [Buquedága enogo]*	antipyretic	preventive remedy; *kido gúru* on the chest; cook the palm heart and drink the cooking water for fever
Unha de vaca	pata-de-vaca, pata-de-boi, unha-de-boi	*Bauhinia forficata* Link., Leguminosae	*Atuboetagureu* [*Atubóe tagaréu*]	anti-inflammatory	preventive remedy; rub leaves on the body
Urucum	colorau, urucu, anatto	*Bixa orellana* L., Bixaceae	*nonógo*	revulsive	

* For updated identifications, visit the webpage *Flora e funga do Brasil*, administrated by Instituto de Pesquisas Jardim Botânico do Rio de Janeiro. Available at: https://reflora.jbrj.gov.br/. Accessed on 17 Mar. 2023; Hartmann (1968, p, p.75-81).

Source: Campos (1913b, 1936); Rondon (1948); Caribé, Campos (1991).

Almost all of them are mentioned by naturalists, who have for a long time traveled through the interior of Brazil. Some, however, are not registered by them, perhaps because of the diversity of common names, often of a purely regional significance. Others involve vague names, to which several plant species are linked (Campos, 1936, p.13).

Certain plants mentioned in the article appear only with their indigenous name, which could mean that the specimen collected was not identified by an expert. According to Hartmann (1968, p.59), much of the difficulty in identifying Bororo medicinal plants originates from the “therapeutic” nomenclature, in which “we encounter the most glaring of discrepancies in the testimony of the informants.” A “nomenclature common to the whole group” seems to coexist with “an almost individual therapeutic nomenclature,” originating from the division that the Bororo make between plants understood to be *erúba* (remedy) and those that are not. This could partly explain Campos’s comment on the lack of uniformity in the treatments prescribed by the *jorubocuros*.

In his presentation, Campos explains that Bororo healers used some medicinal plants for disease prevention (*bôevéboe*), citing their uses and applications:

1. Rub the body with *atugoboétagurêu* (unha de vaca) leaves.

2. Rub the chest with charred *buoquedaga* (tucum).

3. Wash the body with an infusion of *djorubotoboépa* or *betaódjorubo* (*faveira d’anta*).

4. Apply the following to different parts of the body: *kidoguro of ocoïca* (marmelada preta) or *coguridjorubo* (“Cerrado mint”), *sumagaï* (jatobá do campo), and *emaï* (piúva); and *ecoïpo* (souari), *aroécodudireu* (paineirinha); *djorubopagadureu* (herva de passarinho) etc. (Campos, 1936, p.144-145).

Different specific plants were used to treat diseases, which demonstrates the Bororo people’s accumulated knowledge of the Cerrado flora. Campos begins with malaria, specifically how the healers tackled its symptoms, such as fever and heat (*magato, oôrurébôe*, or *boêru*). In general, malaria presented in benign form, but it could take on epidemic proportions if the people were forced to relocate, as described in an outbreak in the Rio das Mortes valley. The remedies used to treat the disease include ingesting an infusion of carobinha do cerrado leaves or an infusion made from the inner bark of the paratudo tree, this latter being recommended for both post-malarial anemia and intestinal hookworm disease (Campos, 1913b, p.114).

Malaria was widespread among the Bororo. Campos diagnosed nine cases among the 81 indigenous people he examined (from the estimated 1,001 individuals living in the five colonies). The movements of troops and scientific expeditions through the Sangradouro military detachment, the São Lourenço telegraph station (set up in 1900), and the recently created SPI post must have exacerbated the spread of diseases among the Bororo (Viveiros, 1958, p.122-123).

Rheumatic disorders were among the most common complaints. At one point, Campos (1936, p.138) attributes these to the Bororo habit of sleeping around a fire on buriti mats and covering themselves with large pieces of fig tree bark. To heal this malady, the healers prescribed ingesting an infusion of erva-molar leaves or rubbing the leaves on painful joints, as well as rubbing the leaves of the sugar apple tree, applying a poultice of the leaves of the paineirinha do campo, and ingesting an infusion of barbatimão leaves (Campos, 1913b, p.114).

Exposure to the cold was also blamed for “flu-like diseases,” which, together with bronchitis, were observed in seven individuals. The *barí* recommended various plant to be made into infusions or rubbed on the body: ingestion of an infusion of *cujarépa* or inhalation of the powdered leaves; ingestion of an infusion of araticum leaves or rubbing of the charred stems on the chest and neck; bathing in an infusion of cruzeirinho; ingestion of an infusion of cambará leaves; and rubbing of araticum leaves on the brow (Campos, 1913b, p.114; 1936, p.146).

Campos (1913b, p.114) defined these bronchopulmonary diseases, which appeared mainly during the dry season, as “frequent and serious” and similarly associated them with the “habit of sleeping by the fire.” They were treated by applying a layer of anatto to the torso and a *quidoguro* or *kido gúru* – a mixture of almecegueira resin and charcoal made from some plants – to the chest, temples, and deltoid regions, which had “revulsive and caustic” action (p.114).

The high number of reported cases of eye conditions (cataracts, conjunctivitis, staphyloma) and blindness led Campos (1913b, p.114) to consider them the veritable “scourge of these Indians,” affecting, as they did, at least 22 individuals, or approximately one quarter of the patients treated. The only settlement where there were no cases was the village of Córrego Grande, while the colony of Sagrado Coração had the highest concentration of conjunctivitis and blindness, as well as all the cases of cataracts (Table 2).

**Table 2:** : Diseases and conditions recorded amongst the indigenous people from the Bororo colonies in eastern Mato Grosso by Murillo de Campos in 1911

Name of disease or condition	C1*	C2*	C3*	C4*	C5*	Total
Malaria	2	-	2	3	2	9
Rheumatism	2	-	-	5	1	8
Conjunctivitis/blindness	3	-	4	9	1	17
Cataract	-	-	-	5	-	5
Staphyloma	1	-	1	-	1	3
Flu/bronchitis	6	-	-	1	-	7
Dysentery	-	-	-	-	1	1
Gastroenteritis	-	-	1	-	-	1
Gastrointestinal disease	-	-	-	1	-	1
Chicken pox	-	-	-	-	3	3
Anemia	-	-	-	2	-	2
Goiter	-	1	-	-	-	1
Scoliosis	1	-	-	-	-	1
Sciatic pain	1	-	1	-	-	2
Dysmenorrhea	-	-	-	2	-	2
Hookworm infection	-	-	-	1	-	1
Scrofula	-	-	-	2	-	2
Toothache	1	-	-	2	-	3
Jigger infestation	-	1	-	-	2	3
Nose ulcer	-	-	-	-	1	1
Others	2	6	-	4	-	12
**Total**	**19**	**8**	**9**	**37**	**12**	**85**

* C1 – São Lourenço colony; C2 – Córrego Grande village; C3 – Barreiros colony; C4 – Sagrado Coração colony; C5 – Sangradouro colony.

Source: Campos (1913b, 1936).

These conditions, known as *iôcochori*, begin as conjunctivitis and may progress over months and years to staphyloma, synechiae, and blindness if not treated and in the case of continued exposure to smoke. Campos (1913b, p.114) attributes this deterioration to the habit of pulling out eyelashes, but he also takes account of the hypothesis provided by a specialist who suspected it was ocular sporotrichosis (Campos, 1936, p.139). The recommended treatment consisted of rinsing the eyes with an infusion of copaiba leaves and applying a poultice of warm *aïgejoruboboecuépa* or *maïa* (unidentified) leaves to the eyelids before going to sleep.

In July 1911, during a visit by the SPI, there was an outbreak of chickenpox in the Sangradouro colony. In fact, epidemics of influenza, scarlet fever, roseola, swamp fever, and yellow fever became relatively frequent among the Bororo in the early twentieth century as a result of the action of missionaries and the Rondon Commission. The concentration of indigenous people in the missions, whether to “defend themselves against attacks from their enemies” or to obtain “treatment in the case of epidemics,” was itself propitious for the “contagion and spread of diseases” (Novaes, 1993, p.149). In 1904, for example, the Salesian colonies were hit by a flu epidemic. The missionaries took advantage of the *bari*’s absence to “administer medicine to the Bororo, assuring them that they would be cured in two days, which in fact happened” (p.147). In 1905, an epidemic of swamp fever took the lives of many people from the Sacred Heart mission, where the indigenous people, despite their increased confidence in Father Giovanni Balzola, continued to turn to *bari* Totó for treatment. In 1913, all the 88 indigenous people who had recently arrived at the Sangradouro mission were stricken with influenza and scarlet fever. Diseases also affected the indigenous people working on the construction of the telegraph line, such as the measles epidemic that broke out in 1901 in the camp on the Água Branca foothills, near Coxim. Rondon even commented on the number of funeral rites and how difficult it was for the Bororo to perform them away from their own lands (Viveiros, 1958, p.132-133).

Prophylactic treatments for “eruptive” diseases would begin by bathing in an infusion of faveira d’anta leaves, applying a *kido gúru*, a poultice prepared from marmelada preta fruits or souari nuts with charred erva de passarinho, to the forehead and deltoids, and rubbing the body with unha de vaca roots. The recommendation for smallpox was an infusion of *pau-terra* bark, while for chickenpox the recommendation was to apply a *kido gúru* of almacega and charcoal from the fruit of the jatobá tree (Campos, 1913b, p.114-115).

One common occurrence was infestation with chiggers (*Tunga penetrans*), found in many patients suffering from a severe rash. They were extracted using a thorn, then *nônôgo* or *nonógo*, a red anatto paste, was spread over the rash or the whole body (Ribeiro, 2006, p.21; Hartmann, 1968, p.61).

During the *marido* ceremony, the buriti dance, which takes place in the middle of the village courtyard, two groups of six men “circle the bones of the dead, carrying on their heads a large disc made of green *buriti* fronds, of an average weight of 9 arrobas, and stamping rhythmically on the ground” (Campos, 1913b, p.115). This great exertion resulted in accidents and even death or heart failure, as Campos witnessed in the São Lourenço colony. To prevent muscle fatigue, it was recommended to tie *bolsa de pastor* leaves with fibers around the ankles and rub the limbs with *chá de frade* leaves.

Despite traveling around the colonies, Campos only recorded one case of goiter. He also remarked on the absence of venereal disease, beriberi, leprosy, tuberculosis, and alcoholism among the Indians, although these were all common in the places he visited. Finally, he did not even find a trace of triatomine bugs in the huts.

## Final considerations

By publishing his medical report as a scientific article, Captain Murillo de Campos was able to expound on the healing practices and ancestral knowledge on medicinal plants of the Bororo Indians of eastern Mato Grosso in greater depth than if he had produced just an official report.

What makes his work original is the care he took in attempting to explain some aspects of the Bororo healing practices. To do so, he relied on *jorubocuros* and other Bororo Indians with whom he came into contact during his stay in the colonies as informants, although at no point did he reveal their identity. Campos’s effort to describe the therapeutic properties and ethnopharmacological indications of the medicinal plants used by shamans, highlighting their indigenous and popular names, as well as some details of how they were used, demonstrates an awareness of the importance of that information and the specimens that could potentially be explored further in a laboratory setting.

In their quest for greater autonomy, especially with the CLTEMTA, military doctors were able to take a more proactive role in the reconnaissance expeditions, assisting scientists from different institutions or carrying out their own fieldwork and making preliminary investigations of the indigenous communities and villages they visited, which gave rise to the ethnographic and scientific work published by the Rondon Commission. This knowledge produced “in transit” through interactions between heterogeneous communities of specialists (doctors and *jorubocuros*) in intercultural contact zones brought about transformations and reconfigurations of the different practices and knowledge.

## Data Availability

Not deposited in a data repository.

## NOTES

1 Linked to the Faculty of Medicine of Rio de Janeiro, it was created in 1887 as a weekly journal promoting national medicine. In the early twentieth century, the focus on sanitation campaigns and efforts to fight epidemics led to a change in the journal’s profile towards medical researchers involved in seeking cures for diseases (Schwarcz, 1993, p.286-287, 293-294).

2 The Service for the Protection of Indians and Location of National Workers (Serviço de Proteção aos Índios e Localização de Trabalhadores Nacionais, SPI) began to operate in 1910 under the direction of Cândido Rondon, who devised it as an offshoot of the CLTEMTA designed to settle and group together indigenous people as part of efforts to develop a national labor force (Maciel, 1998, p.171).

3 [Translator’s note] Citations in English from Latour (2000) were sourced from Bruno Latour, *Science in action: how to follow scientists and engineers through society*, Cambridge, MA, Harvard University Press, 1987.

4 This indigenous group, known as Otuque, Bororo, or Boe (self-denomination), still live in the east of this state. They speak the Bororo language, which they call Boe Wádaru, which may belong to the Macro-Gê family of languages (Urquiza, 2007).
